# The MicroRNA319d/TCP10 Node Regulates the Common Bean – Rhizobia Nitrogen-Fixing Symbiosis

**DOI:** 10.3389/fpls.2018.01175

**Published:** 2018-08-10

**Authors:** José Á. Martín-Rodríguez, Alfonso Leija, Damien Formey, Georgina Hernández

**Affiliations:** Centro de Ciencias Genómicas, Universidad Nacional Autónoma de México, Cuernavaca, Mexico

**Keywords:** microRNAs, legume–rhizobia interaction, symbiotic nitrogen fixation, nodules, common bean, *Phaseolus vulgaris*

## Abstract

Micro-RNAs from legume plants are emerging as relevant regulators of the rhizobia nitrogen-fixing symbiosis. In this work we functionally characterized the role of the node conformed by micro-RNA319 (miR319) – TEOSINTE BRANCHED/CYCLOIDEA/PCF (TCP) transcription factor in the common bean (*Phaseolus vulgaris*) – *Rhizobium tropici* symbiosis. The miR319d, one of nine miR319 isoforms from common bean, was highly expressed in root and nodules from inoculated plants as compared to roots from fertilized plants. The miR319d targets *TCP10* (Phvul.005G067950), identified by degradome analysis, whose expression showed a negative correlation with miR319d expression. The phenotypic analysis of *R. tropici*-inoculated composite plants with transgenic roots/nodules overexpressing or silencing the function of miR319d demonstrated the relevant role of the miR319d/TCP10 node in the common bean rhizobia symbiosis. Increased miR319d resulted in reduced root length/width ratio, increased rhizobial infection evidenced by more deformed root hairs and infection threads, and decreased nodule formation and nitrogenase activity per plant. In addition, these plants with lower TCP10 levels showed decreased expression level of the jasmonic acid (JA) biosynthetic gene: *LOX2.* The transcription of *LOX2* by TCPs has been demonstrated for Arabidopsis and in several plants *LOX2* level and JA content have been associate with TCP levels. On this basis, we propose that in roots/nodules of inoculated common bean plants TCP10 could be the transcriptional regulator of *LOX2* and the miR319d/TCP10 node could affect nodulation through JA signaling. However, given the complexity of nodulation, the participation of other signaling pathways in the phenotypes observed cannot be ruled out.

## Introduction

Legumes are ecologically important because of their ability to establish an efficient symbiotic association with nitrogen-fixing rhizobia, resulting in the formation of root nodules, where rhizobia can fix the atmospheric dinitrogen (N_2_) in forms that can be assimilated by the plant, in exchange for a carbon source. Symbiotic nitrogen fixation (SNF) reduces the cost of legume cultivation and is relevant for sustainable agriculture (Venkateshwaran et al., 2013). The evolution of this symbiosis was a key to success for the legume family that comprises 18,000 described species with approximately 700 genera and represents one-third of the primary crop production in the world; however, legume production necessary for feed and food relies on only a few cultivated species (Doyle and Luckow, 2003). *Phaseolus vulgaris*, known as common bean, is the principal source of non-animal protein for human consumption in the developing world (Broughton et al., 2003). Besides the caloric and proteic intake, common bean grains have high contents of fiber, complex carbohydrates and other dietary elements as minerals, thiamine, folate, and a variety of flavonoids and secondary metabolites with medicinal properties (Blair et al., 2013).

In recent years, several studies have shown different classes of small non-coding RNAs (sRNA) that act as essential regulators of gene expression in plants. MicroRNAs (miRNA) are a major class of sRNA, 21 – 24 nt in length, that regulate gene expression post-transcriptionally through sequence complementarity, either via target transcript cleavage or translational inhibition. Plant miRNAs are involved in most, if not all, biological processes such as development, hormone regulation, nutrient homeostasis and interaction with pathogens and symbionts (reviewed by Jones-Rhoades and Bartel, 2004; Rogers and Chen, 2013; Li et al., 2017). Growing evidence supports the participation of miRNAs in the control of the legume-rhizobia symbiosis (Lelandais-Brière et al., 2016). Studies based on high-throughput sRNA sequencing have identified miRNA families that are expressed in nodules from different legume species (Subramanian et al., 2008; Lelandais-Brière et al., 2009; De Luis et al., 2012; Turner et al., 2012). For common bean we identified a set 185 mature miRNAs, 106 of this, including 50 previously unpublished sequences, were present in nodules (Formey et al., 2015). Aiming to understand the role in nodules of newly identified common bean miRNAs we constructed weighted correlation networks of miRNAs with differential expression in the nodule library as compared to other libraries. The networks include miRNAs known to play regulatory roles in nodules suggesting a similar role for novel miRNAs (Formey et al., 2015). One of these weighted correlation networks included an isoform of the miR319 family, pvu-miR319d, initially described in soybean (Wong et al., 2011). In this work we analyzed the role of miR319d from common bean in symbiosis with *R. tropici*.

Though the majority of plant miRNAs have been identified by large-scale sequencing strategies and bioinformatics approaches based on the conservation of fold-back precursors (Jones-Rhoades and Bartel, 2004), Arabidopsis miR319a is an exception as it was isolated through the screening of an activation-tagging T-DNA transgenic population that generated dominant gain-of–function mutations (Weigel et al., 2000). The first described plant miRNA mutant, *jaw*-D, overexpresses miR319a that is one of the first characterized and conserved plant miRNA families. It was demonstrated that the conserved miR319 targets are the plant-specific transcription factors (TF) *TCP* (for *TEOSINTE BRANCHED/CYCLOIDEA/PCF*) (Palatnik et al., 2003). The TCP domain codes a DNA-binding motif that folds into a basic helix-loop-helix structure (Cubas et al., 1999). The TCP TFs participate in various important aspects of plant development, especially the control of cell division, expansion, and differentiation during leaf development but also other important functions such as mitochondrial biogenesis, leaf senescence and floral development (Palatnik et al., 2003; Schommer et al., 2008, 2012; Martín-Trillo and Cubas, 2010). The TCPs can be subdivided in two main branches (class I and II) according to their sequence in the TCP domain. In Arabidopsis, the TCPs comprise a family of 24 members; only five of these (TCP2, TCP3, TCP4, TCP10, and TCP24) belonging to class II are targets of miR319 (Palatnik et al., 2003; Schommer et al., 2008).

The networks of TFs regulated by miRNAs can interact with others during plant development (Rubio-Somoza and Weigel, 2011). Several studies have revealed the interaction of the miR319/TCP node with miR164 and miR396 (Schommer et al., 2012). In Arabidopsis TCPs, belonging to class II, directly activate the transcription of *MIR396*; this miRNA targets *GROWTH-REGULATING FACTORS* (*GRF*) TFs that in turn regulate cell proliferation via the control of cell cycle genes (Rodríguez et al., 2010).

The leaf morphogenesis process that is regulated by the miR319/TCP node has been linked with other processes such as jasmonic acid (JA) biosynthesis and senescence (Schommer et al., 2012). JAs are lipid-derived signaling molecules in plants that regulate diverse responses to wounding, pathogen attack, reproduction, development, metabolic regulation and abiotic stress (Devoto and Turner, 2003; Howe, 2004). The participation of JA in the legume-rhizobium symbiosis has been reported in several studies (Sun et al., 2006; Seo et al., 2006; Poustini et al., 2007; Ferguson and Mathesius, 2014). However these studies are yet inconclusive, collectively these appear to indicate that JAs can act as either positive or negative regulators of nodulation and nitrogen fixation, depending on the legume species, the type of JA used, and when, where, and how the hormone is applied (Ferguson and Mathesius, 2014). The first dedicated step in the biosynthesis of JA is catalyzed by lipoxygenases encoded by the *LOX* genes. In Arabidopsis *LOX2*, and other three *LOX* genes, encode chloroplast-localized lipoxygenases that catalyze the conversion of α-linolenic acid (18:3) into (13S)-hydroperoxyo-linolenic acid. The *LOX2* is one of the most affected genes in the transcriptome of *tcp* loss-of-function Arabidopsis mutants (Schommer et al., 2012). It has been demonstrated that Arabidopsis TCPs recognize specific binding sites present in the *LOX2* promoter to directly regulate its transcription (Schommer et al., 2008; Danisman et al., 2012). Other JA biosynthetic genes also respond to miR319/TCP levels include the *ALLENE OXIDE SYNTHASE* (*AOS*) that catalyzes the conversion of 13-hydroperoxy-linolenic acid to an unstable allene oxide intermediate (Schommer et al., 2008; Zhang et al., 2016).

To our knowledge the participation of the miR319/TCP node as regulator of the legume – rhizobia symbiosis has not been reported. In this paper we analyzed the role of the common bean miR319d isoform and its target TCP10 in the symbiosis with *Rhizobium tropici*. We confirmed the high expression of miR319d in roots/nodules of SNF common bean as compared to tissues from fertilized (non-inoculated) plants. The functional analysis of composite common bean plants with modulated expression of this miRNA revealed the effect of the miR319d/TCP10 node in root development, rhizobia infection, nodulation and SNF. These effects could be related with observed alterations in the expression of *LOX2*, a JA biosynthetic gene, and the participation of JA in the regulation of different stages of symbiosis with *Rhizobium*.

## Materials and Methods

### Phylogenetic Analysis

miR319 isoform sequences from *Phaseolus vulgaris* were obtained from the small RNAseq analysis performed by Formey et al. (2015), where each miR319 isoform was referred as designated in the plant species it was discovered. The **Supplementary Table S1** shows the equivalence among the nomenclatures from this work and those from Formey et al. (2015) for each *P. vulgaris* miR319 isoform sequence.

TCP protein sequences were obtained from the *Phaseolus vulgaris* release v2.1, from Phytozome 12 database^1^. Sequence alignments were performed thanks to MAFFT online service v7 (Katoh et al., 2017) with L-INS-i option set. Construction of phylogenetic tree of miR319 isoforms and TCP protein sequences were based on the average linkage (UPGMA) method and Neighbor-Joining JTT model, respectively. Bootstrap values were obtained after 100 resampling.

### Plasmid Construction, Plant Transformation and Generation of Composite Plants

The overexpression and silencing of miR319d function in common bean transgenic roots were carried out using the pTDTO plasmid (Aparicio-Fabre et al., 2013). This expression plasmid contains the 35S cauliflower mosaic virus (35SCaMV) promoter and the tdTomato (red fluorescent protein) gene as a visible reporter gene. The precursor of miR319d (286-bp) was PCR-amplified using as template cDNA from common bean nodules and the specific primers Fw-pre319d (5′-ATGGATCCTGATACTAGAGTACAGGGAGA-3′) and R-pre319d (5′-TCTCGAGTTGTGTGTATGTATTAATATTAATG-3′). To silence miR319 function the “Short Tandem Target Mimicry” (STTM) method (Yan et al., 2012) was employed using the specific primers Fw-STTM319d (5′-ATGGATCCGAAGGAGCTCCCTACCTTCAGTCCAGTTGTTGTTGTTATGGTCTAATTTAAATATGGTC-3′) and R-STTM319d (5′-ACTCGAGTGGACTGAAGGTAGGGAGCTCCTTCATTCTTCTTCTTTAGACCATATTTAAATTAGACC-3′). The purified PCR products were cleaved by *Xho*I and *BamH*I sites and cloned into the pTDTO expression vector. The empty vector pTDTO, hereafter denominated EV, and the resulting OEmiR319d and STTMmiR319d plasmids were introduced by electroporation into *Agrobacterium rhizogenes* K599, which was then used for plant transformation as described previously (Estrada-Navarrete et al., 2007) with minor modifications (Aparicio-Fabre et al., 2013). The presence of red fluorescence resulting from the of the tdTomato reporter gene was routinely checked in the putative transgenic roots using light microscopy.

### Plant Material and Growth Conditions

The common bean (*P. vulgaris* L.) Mesoamerican cv BAT 93 was used in this work. Seeds were surface sterilized in 10% (v/v) commercial sodium hypochlorite for 5–10 min and finally rinsed 5–6 times in sterile distilled water. Subsequently seeds were germinated on moist sterile paper towels at 30°C for 2–3 days in darkness. Germinated seedlings of similar size were planted in pots with wet sterile vermiculite. After 2 days of adaptation plants were inoculated with 1 ml saturated liquid culture of the *Rhizobium tropici* CIAT 899 strain per plant. Plants were grown in growth chambers under controlled environmental conditions (25–28°C, 16 h photoperiod) and were watered every 3 days with N-free B&D nutrient solution (Broughton and Dilworth, 1971). For fertilized and non-inoculated condition, full nutrient B&D solution was used to water the plants. Common bean composite plants with transgenic roots were generated as described below and grown in similar conditions to those for wild-type plants. Plants were harvested at different time points for analysis; tissues for RNA isolation were collected directly into liquid nitrogen and stored at -80°C.

### RNA Isolation and Analysis

Total RNA was isolated from 100 mg tissues using mirVana^TM^ miRNA Isolation Kit (Ambion) following the supplier’s recommendations. For *R. tropici*-inoculated BAT 93 plants, the tissues used for RNA isolation were roots separated from nodules and detached nodules. For *R. tropici*-inoculated composite plants RNA was isolated from transgenic nodulated root system. Three samples (biological replicates) for each tissue from different plants grown under similar conditions were analyzed.

For the quantification of mature miRNA transcript accumulation levels, cDNAs were prepared using RevertAid reverse transcriptase (Fermentas) following the stem-loop method (Kramer, 2011). Stem-loop primers for reverse transcription of miRNAs were designed as reported by Chen et al. (2005). The conditions used were: denaturation at 65°C for 5 min, then 16°C for 30 min; 60 cycles of 30°C for 30 s, and 42°C for 30 s, 50°C for 1 s followed by 70°C for 15 min. Primers for qRT-PCR amplification are listed in **Supplementary Table S2**. Resulting cDNAs were then diluted 10-fold and used to perform the qRT-PCR experiments using SYBR Green qPCR Master Mix (Fermentas) following manufacturer’s instructions. The reaction mix was then dispensed in a 96 well plate and analyzed using real-time thermocycler Applied Biosystem 7300 (Foster City, CA, United States). The thermal cycler settings were as follows: 94°C for 1 min, followed by 40 cycles of 94°C for 20 s and 60°C for 60 s. Relative transcript levels for each sample were obtained using the ‘comparative C_t_ method’ and normalized with the geometrical mean of three housekeeping genes (*HSP, MDH*, and *UBQ9*) (Vandesompele et al., 2002) and the U6 sRNA, for the mRNA transcripts and the miRNAs, respectively. In all of our qRT-PCR analyzes a well-defined melting curve was obtained both for miRNAs and for cDNAs. A Mann-Whitney statistical test was performed to evaluate the significance of the differential expression using the mean values from three biological replicates for each condition, using the GraphPad Prism program.

### Phenotypic Analysis

Nitrogenase activity was determined in detached nodulated roots form composite plants by the acetylene reduction assay essentially as described by Hardy et al. (1968). Specific activity is expressed as nmol ethylene h^-1^ per plant. The root fresh weight, area, length and root width were determined in composite plants grown, for 24 dpi, under symbiotic conditions. The quantification of root hair deformation and induction of infection thread upon rhizobial inoculation was performed in samples from the responsive zone of roots inoculated with *R. tropici* CIAT 899 for 2 or 6 days. Samples were collected into PBS buffer and were stained with 0.01% (w/v) Methylene Blue for 1 h and washed three times with double-distilled water. Infection events were observed in the optical microscope Axioskop 2 (Zeiss), at least 5 different root responsive zone samples (biological replicates) were used for analysis. Statistical analyses were performed using the Mann–Whitney null hypothesis statistical test.

### Prediction of Transcription Factors Binding Sites (TFBS)

To predict the transcription factors that could regulate the *LOX2* and *LOX5* genes, we performed an analysis using Clover (pre-2010 version, Frith et al., 2004) and the plant JASPAR CORE motif library (Sandelin and Wasserman, 2004) on the 4 kb upstream sequence of 5′UTR end of the corresponding genes. Predicted motifs with a *p*-value > 0.05 were discarded.

## Results

### Common Bean miR319 Isoforms and Target Genes

**FIGURE 1:**
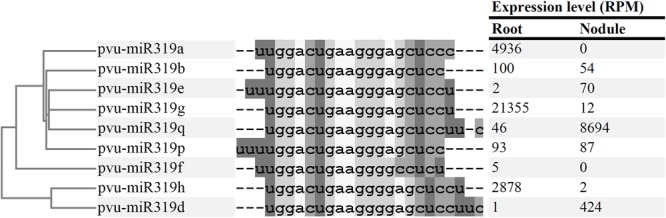
Alignment of mature microRNA sequences from *Phaseolus vulgaris* miR319 family. **(Left)** Panel contains a tree representing the phylogenetic relationship between the miRNA sequences. **(Central)** Panel contains the aligned microRNA sequences. Dashes represent the mismatches of the alignment. **(Right)** Panel is a table containing the expression level (RPM) of each microRNA in the Root and Nodule libraries published in Formey et al. (2015).

The *Arabidopsis* genome contains three loci that generate miR319 isoforms ath-miR319a to ath-miR319c, while seventeen miR319 isoforms are reported for soybean (*Glycine max*) (v.22)^2^. The high-throughput small RNA (sRNA) sequencing analysis by Formey et al. (2015) identified nine miR319 isoforms in common bean. Of these, five isoforms have been identified in soybean, a legume related to common bean and one was identified in grape (*Vitis vinifera*) (v.22)^2^, while four are new miR319 isoforms similar to the soybean isoforms gma-miR319c, d or f. Initially their nomenclature referred to the species where each miR319 isoforms were identified, while in this work we propose the pvu-miR nomenclature for the *P. vulgaris* miR319 isoforms (**Figure 1** and **Supplementary Table S1**). The common bean miR319 isoforms, 18–22 nucleotides long, showed sequence similarities and are grouped into two well-differentiated clades (**Figure 1**). One clade included mature miRNAs with four guanosines in their central region nucleotide sequence while in the other clade only three guanosines were observed. Each clade grouped two of the novel common bean miR319 isoforms.

Analyses of sRNA-seq data from libraries generated from different plant organs have identified conserved and legume-specific miRNA families differentially expressed during nodule organogenesis in different legumes (De Luis et al., 2012; Turner et al., 2012; Formey et al., 2014). Our previous reports (Formey et al., 2015) revealed three isoforms of common bean miR319 with higher expression level in nodules as compared to roots: pvu-miR319e (35-fold), pvu-miR319q (189-fold) and pvu-miR319d (424-fold) (**Figure 1**). In addition to showing the highest nodule/root expression ratio, pvu-miR319d isoform was included by Formey et al. (2015) in a weighted correlation network of common bean miRNAs with significantly increased expression in the nodule library as compared to other libraries. These features lead us to select the common bean pvu-miR319d isoform for this study aiming to characterize its regulatory role in the rhizobia symbiosis. The pvu-miR319d, hereafter referred as miR319d, was initially identified via high-throughput sequencing data and annotated in the soybean miRBase database by Wong et al. (2011). In common bean the gene encoding miR319d was mapped to chromosome 9 (nucleotides 8534451-8534637), it generates a 187-nucleotides pre-miRNA with *bona fide* stem-loop secondary structure that give rise to the 22-nucleotides mature miRNA encoded close to the 3′ end of pre-miR319d (Formey et al., 2015).

**FIGURE 2:**
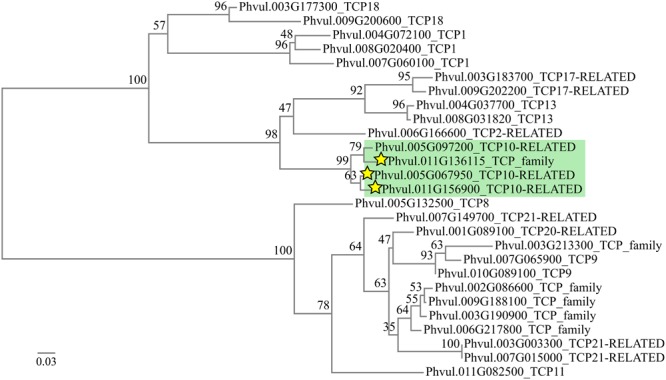
Phylogenetic tree of 27 TCP sequences from *Phaseolus vulgaris*. Numbers at the branch dichotomy represent the bootstrap values obtained after 100 resampling. The green box contains the transcripts predicted as miR319 targets. Stars indicate the transcripts found as degraded by a member of the miR319 family in the degradome data from Formey et al. (2015). Scale bar represents the branch length.

The conserved targets for miR319 in different plants are transcripts that encode transcription factors (TF) of class II subclass of the TCP family. Of the 24 *Arabidopsis TCP* TF genes, five contain a target site for miR319 (*TCP2, TCP3, TCP4, TCP10*, and *TCP24*) that, in every case, is located outside the TCP domain and near the 3′ part of the coding region (Schommer et al., 2012). In soybean, 14 *TCP* TF genes have been proposed as miR319 targets (Song et al., 2011; Goettel et al., 2014). Sequence analyses from genomic and transcriptomic data (O’Rourke et al., 2014; Schmutz et al., 2014), led us to identify 27 *TCP* TF genes for common bean (**Figure 2**). From the whole set (27) we have identified 4 *TCP* genes with putative miR319 binding sites near their 3′ part of their coding sequence (Formey et al., 2015). Interestingly, the TCP predicted targets of miR319 were organized in a single clade of the phylogenetic tree (**Figure 2**). From these, Phvul.011G136115, Phvul.005G067950 and Phvul.011G156900 were identified as targets in a degradome analysis (Formey et al., 2015). The base pairing of each predicted TCP target gene with the miR319d isoform as well as their expression level in roots and nodules, (v12.1.6, *Phaseolus vulgaris* v2.1)^3^ are shown in **Table 1**. Phvul.005G097200 and Phvul.011G136115 transcripts showed several mismatches thus a high penalty score for miRNA:mRNA pairing (**Table 1**) that would not fulfill the requirements for a miR319d target according to Jones-Rhoades and Bartel (2004). Phvul.011G136115 was not expressed and Phvul.005G097200 showed similar expression in roots and nodules (**Table 1**). Contrastingly, Phvul.005G067950 and Phvul.011G156900 transcripts showed similar low penalty score, a perfect base pairing in the 5′ miRNA region and a hybridization energy value of -41.05 Kcal/mol (Jones-Rhoades and Bartel, 2004; Hammell et al., 2008). The root/nodule expression profile was different among these transcripts, Phvul.011G156900 showed low expression level and slightly higher in nodules while Phvul.005G067950 showed higher expression level in roots than in nodules (**Table 1**). Combined together, these results converge toward the fact that miR319d induces the cleavage of its target Phvul.005G067950. On this basis, we selected this *TCP* gene, hereafter denominated *TCP10*, as the target of miR319d for the analysis of this node as possible regulator in the common bean – rhizobia symbiosis. However, the targeting of miR319d to Phvul.011G156900 in other plant organs or growth conditions cannot be ruled out.

**Table 1:** Common bean TCP transcripts with miR319 binding sites.

Gene name	Alignment (penalty score)	Expression (FPKM)	
		Roots	Nodules
pvu-miR319d	22 CUUCCUCGAGGGGAAGUCAGGU 1		
	: . : . : : : : : : : : : : : : : :		
Phvul.005G067950	1362 CAGAGGGGACCCCUUCAGUCCA 1383 (3.5)	16	8

pvu-miR319d	22 CUUCCUCGAGGGGAAGUCAGGU 1		
	: . : . : : : : : : : : : : : : : :		
Phvul.0llGl56900	1974 CAGAGGGGACCCCUUCAGUCCA 1995 (3.5)	2	3

pvu-miR319d	22 CUUCCUCGAGGGGAAGUCAGGU 1		
	: : . : . : . : : : : : : : :		
Phvul.005G097200	1317 CUUGGCCUUUCUCUUCACUCCU 1338 (6.0)	11	11

pvu-miR319d	22 CUUCCUCGAGGGGAAGUCAGGU 1		
	: : : : : : : : : : : : : : : :		
Phvul.011G136115	1533 CAAAGUGAGACCCUUCAGUCCA 1554 (7.5)	0	0

*Pairing of four TCP putative target genes identified with a miR319 binding site (Formey et al., 2015). Watson-Crick base pairing is indicated by two dots, G-U base pairing by one dot and mismatches are empty. Penalty scores shown in parenthesis were calculated as described by Jones-Rhoades and Bartel (2004). Expression values (FPKM, Fragments Per Kilo Million) from *Phaseolus vulgaris* release v2.1 from Phytozome 12 database, are shown.*

### Expression Analysis of miR319d and TCP10 During Rhizobia Symbiosis

The role of miR319 in leaf/shoot development has been well documented for Arabidopsis and other plants (Schommer et al., 2012, 2014; Koyama et al., 2017). However, although miR319 isoforms have been identified in roots from different plants, its possible regulatory role in this organ has not been extensively studied. There are some reports on miR319 response to heavy metals, to ethylene or to pathogens in roots of different plants (Valdés-López et al., 2010; Chen et al., 2012; Li et al., 2012; He et al., 2014).

**FIGURE 3:**
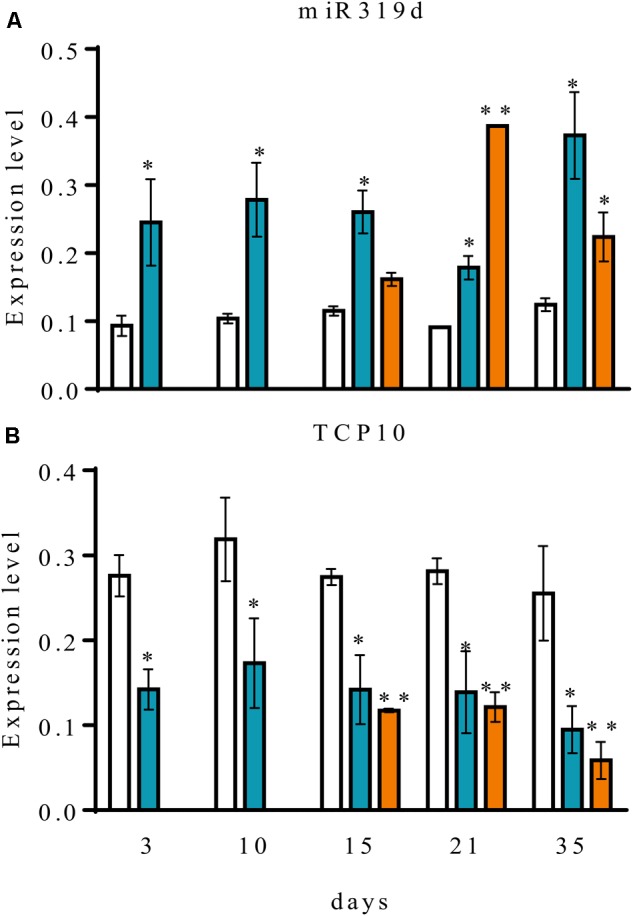
Expression analysis of common bean miR319d and TCP10 target gene in roots (blue histograms) and nodules (orange histograms) of *R. tropici* CIAT899-inoculated plants and root (white histograms) of fertilized (non-inoculated) plants. Expression level of mature miR319d **(A)** and its target gene *TCP10* **(B)** were determined by qRT-PCR in inoculated roots or nodules harvested at the indicated time points, corresponding to days post inoculation for inoculated plants or days after planting for fertilized plants. Expression level refers to gene expression, based on Ct value, normalized with the expression of the housekeeping *U6* for miR319d and *UBC9, HSP* and *MDH* for *TCP10* gene. Values represent means ± SD from three biological replicates and two technical replicates each. The Mann-Whitney null hypothesis statistical test is relative to data from fertilized plants from the same harvest (^∗^ and ^∗∗^ represent a *p-*value < 0.05 and *p-*value < 0.01, respectively).

To assess the possible role of common bean miR319d/TCP10 in the rhizobia symbiosis we performed a qRT-PCR expression analysis in roots separated from nodules and in detached nodules of *R. tropici*-inoculated common bean plants at different stages of development. The expression level of miR319d/TCP10 in roots from fertilized (non-inoculated) plants was included for comparison (**Figure 3**). The different developmental stages of root and nodules from inoculated plants could be defined by the differential expression of an early-nodulin gene and with the level of nitrogenase activity (**Supplementary Table S3**). *ENOD40* (Phvul.002G064166), which lacks an open reading frame but encodes two small peptides and may function as a cell-cell signaling molecule for nodulation (Wang et al., 2014) showed a very low expression in fertilized roots that contrasts with its increased transcript level in inoculated roots at early stages (3 dpi) and the level persists until decreasing in senescent nodule (35 dpi). Also, *ENOD40* showed a high expression in nodules, especially in immature nodules (15 dpi). Nitrogenase showed its highest activity in mature (21 dpi) nodules as compared to immature (15 dpi) and senescent (35 dpi) nodules (**Supplementary Table S3**). As shown in **Figure 3A**, the fertilized root showed a low expression level of miR319d, that remains constant in the different developmental stages. In contrast, the inoculated roots showed significantly increased expression of miR319d at all stages. In nodules, miR319d expression level varied according to its developmental stages being low in immature nodules, increasing (*ca*. 2-fold) in mature nodules and slightly decreasing in senescent nodules (**Figure 3A**).

We determined the transcript levels of the miR319d target gene *TCP10* in similar tissues from fertilized and inoculated common bean plants (**Figure 3B**). *TCP10* transcript level was high in fertilized roots and it remained constant during the different time points. However *TCP10* expression level in roots/nodules from inoculated plants was significantly lower, reaching the lowest values in senescent nodules. The data obtained by qRT-PCR expression analysis validated those previously reported from RNA-seq data analysis (O’Rourke et al., 2014; Formey et al., 2015). Overall, a negative correlation was observed between the miR319d vs. *TCP10* expression levels in fertilized as compared to inoculated plants (**Figures 1, 3** and **Table 1**).

To complete our study, we analyzed the expression of the Phvul.011G156900 *TCP* gene an alternative putative target of miR39d (**Table 1**). The expression analysis of this *TCP* gene, that validated previous data^4^, revealed low values, up to 60-fold lower as compared to those for *TCP10*; the values did not vary among the different tissues and time points tested for inoculated and fertilized plants (**Supplementary Figure S1**). In addition, no negative correlation between Phvul.011G156900 and miR319d expression was observed. These data support our analysis of *TCP10* as the target gene of miR319d in common bean roots/nodules.

### Effect of the Modulation of miR319d Expression on Root Development and Rhizobial SNF

**FIGURE 4:**
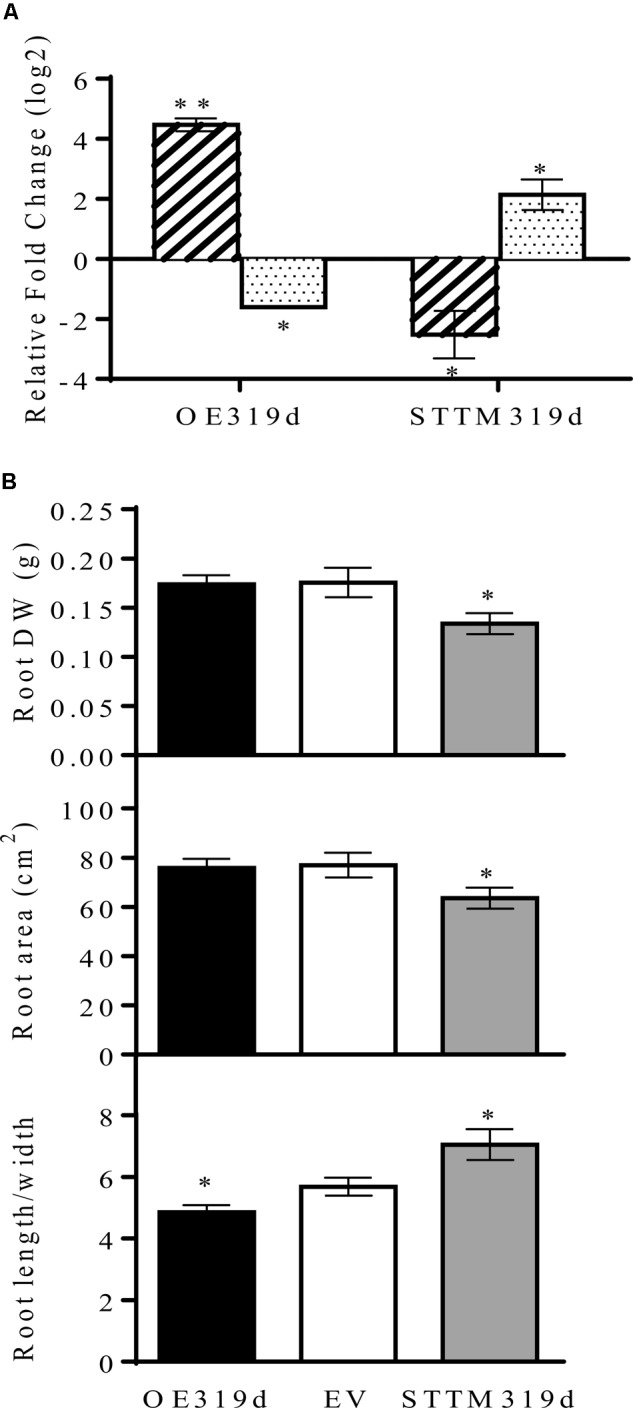
miR319d and TCP10 affect root morphology of *R. tropici*-inoculated common bean plants. **(A)** Transcript levels of mature miR319d (diagonally striped histograms) and *TCP10* (dotted histograms) were determined by qRT-PCR in 24 dpi inoculated roots of composite plants transformed with OE319d or STTM319d constructs. Values (log_2_) were normalized to the value from control or EV-transformed inoculated roots that was set to 0. Values represent means ± SD from three biological replicates and two technical replicates each. **(B)** Roots dry weight (DW), area and length/width ratio from composite bean plants with transgenic roots overexpressing (OE319d, black histograms) or silencing miR319d (STTM319d, gray histograms) as compared with control roots (EV-transformed, white histograms). Values represent means ± SD from nodulated roots of sixteen to twenty independent composite plants each. The Mann–Whitney null hypothesis statistical test is relative to EV control data (^∗^ and ^∗∗^ represent a *p-*value < 0.05 and *p-*value < 0.01, respectively).

To further investigate the role of miR319d/TCP10 in the common bean – rhizobia SNF, we aimed to modulate the miR319d expression in common bean composite plants with transgenic roots and untransformed aerial organs, generated through *A. rhizogenes*-mediated genetic transformation (Estrada-Navarrete et al., 2007). This protocol, used as an alternative method for stable transformation in common bean and other recalcitrant legume species, has been successfully used by our group to demonstrate miRNA functionality (Valdés-López et al., 2008; Naya et al., 2014; Nova-Franco et al., 2015). The construct for overexpression of the miR319d precursor (OE319d) and the function silencing function construct (STTM319d) (Yan et al., 2012) were driven by the 35SCaMV promoter. Both constructs as well as the control empty vector (EV) contained the tdTomato (red fluorescent protein) reporter gene (Naya et al., 2014). The transcript level of miR319d and *TCP10* from transgenic roots of rhizobia-inoculated composite plants transformed with OE319d and STTM319d constructs, normalized to the transcript level values from EV transgenic roots, are shown in **Figure 4A**. As expected, the OE319d composite plants showed very high level of miR319d and a decreased level of *TCP10* transcript. Conversely, the STTM319d plants showed low levels of miR319d and increased *TCP10* transcript levels. Also, we determined the transcript levels of the Phvul.011G156900 *TCP* gene, the alternative putative target of miR39d (**Table 1**), the values in OE319d and STTM319d transgenic roots were not significantly different from those of EV (**Supplementary Figure S1**). These data again indicate that *TCP10* (Phvul.005G067950), and not Phvul.011G156900, is the target gene of miR319d in common bean roots/nodules.

We first assessed if the modulation of miR319d expression affects the root phenotype of *R. tropici*-inoculated common bean plants. As compared to control EV roots, the roots with low miR319d (STTM319d) showed decreased root biomass and area as well as higher length/width ratio due to longer and less dense roots (**Figure 4B** and **Supplementary Figure S2**). By contrast, the OE319d roots showed lower length/width ratio and similar root biomass and area as compared to control roots (**Figure 4B**).

**FIGURE 5:**
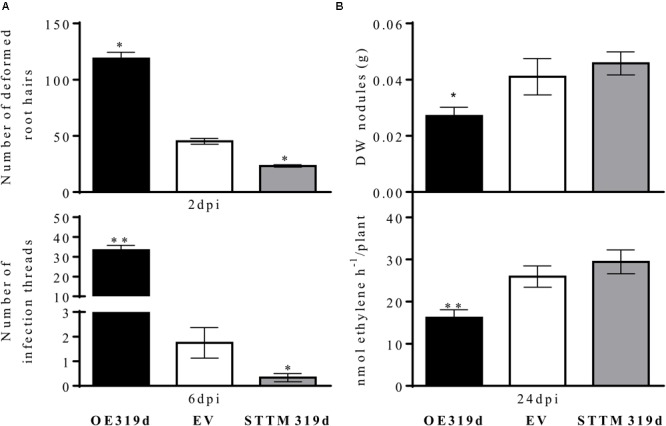
miR319d and TCP10 control the rhizobial infection, nodulation and nitrogen fixation of *R. tropici*-inoculated common bean plants. **(A)** At 2 and 6 dpi, the responsive zone of OE319d, EV or STTM319d inoculated roots were harvested for quantification of the number of deformed root hairs (branched and swollen root hairs) and of infection threads per 0.5 cm, respectively. **(B)** Dry weight of nodules and nitrogenase activity were determined at 24 dpi. Black histograms: OE319d, gray histograms: STTM319d, EV white histograms. Values represent means ± SD from roots of 10 to 20 independent composite plants each. The Mann–Whitney null hypothesis statistical test is relative to EV control data (^∗^ and ^∗∗^ represent a *p-*value < 0.05 and *p-*value < 0.01, respectively).

To analyze if the effect of miR319d on root development also affects rhizobial infection and SNF, we investigated the response of miR319d-modulated composite plants to *R. tropici* CIAT 899 infection, nodulation and SNF. Regarding rhizobial infection, we quantified the root hair deformation and the infection thread formation at early symbiotic stages. Notably, the amount of deformed root hairs was significantly higher in 2 dpi-inoculated roots that over-express miR319d, while the opposite effect was observed in STTM319d roots (**Figure 5A**). In agreement with this result, 6 dpi-inoculated OE319d roots showed a high increase in the amount of infection threads and the opposite effect was observed in STTM319d inoculated roots (**Figure 5A**). In addition, earlier infection thread formation, at 2 dpi, was observed only in OE319d roots and not in the other composite plants (**Supplementary Figure S3**). At nodule maturation (24 dpi) OE319d composite plants showed lower nodule biomass and nitrogenase activity as compared to EV and STTM plants (**Figure 5B**). Nodule biomass correlated with nodule number and not with altered nodule size because the nodule perimeter was similar in nodules from the different composite plants (OE319d = 0.43 ± 0.011 mm, EV = 0.43 ± 0.018 mm, STTM319d = 0.43 ± 0.018 mm). Overall, miR319d-overexpressing composite plants, with low TCP10 content (**Figure 4A**), showed a different pattern of effects in rhizobial infection vs. nodule formation/SNF (**Figures 5A,B**).

### Exploring Downstream TCP10 Regulation in OE319d and STTM319d SNF Plants

The TCP class II TF, targets of miR319, participate in complex regulatory networks that coordinate and balance different events that are important for plant development and physiology. Relevant functions of TCP genes are the control of leaf and flower size and shape. The signaling pathways associated with these functions include, among others, the regulation of leaf cell proliferation by *GROWTH REGULATING FACTOR* (*GRF*) TFs, targets of miR396, and also the biosynthesis of JA that regulates different processes such as senescence (Nicolas and Cubas, 2016).

On this basis, we first analyzed if *TCP10* activates the transcription of *MIR396*, resulting in the degradation of *GROWTH REGULATING FACTOR* (*GRF*) induced by mature miR396 to control cell proliferation in common bean inoculated-roots. We quantified the mature miR396 transcript accumulation levels in OE319d and STTM319d nodulated roots, with diminished and increased TCP10 levels, respectively. Our data showed similar levels of miR396 in roots form the different composite plants analyzed (**Supplementary Figure S4**) thus indicating that TCP10 does not regulate miR396 transcription. We propose that miR319d/TCP10 node is not involved in the regulation of the miR396/GRF node nor in the proliferation of cells from common bean roots/nodules. However, further work is required to define if other common bean miR319 isoforms (**Figure 1**) that are highly expressed in leaves, such as pvu-miR319g and pvu-miR319h (Formey et al., 2015) and their TCP target genes participate in the miR396/GRF regulatory network to control leaf development.

The miR319/TCP regulation of leaf morphogenesis has been linked to JA biosynthesis (Schommer et al., 2012). It has been demonstrated that, two Arabidopsis TCP TFs bind to the *LOX2* gene promoter and directly regulate its transcription (Schommer et al., 2008; Danisman et al., 2012). In addition, the expression of other JA-biosynthetic and -responsive genes depends on miR319/TCP levels in several plant species (Schommer et al., 2008; Danisman et al., 2012; Hao et al., 2012; Zhang et al., 2016, 2017). On this basis, we analyzed a possible correlation of TCP10 levels and the expression level of JA-related genes in common bean roots /nodules.

**FIGURE 6:**
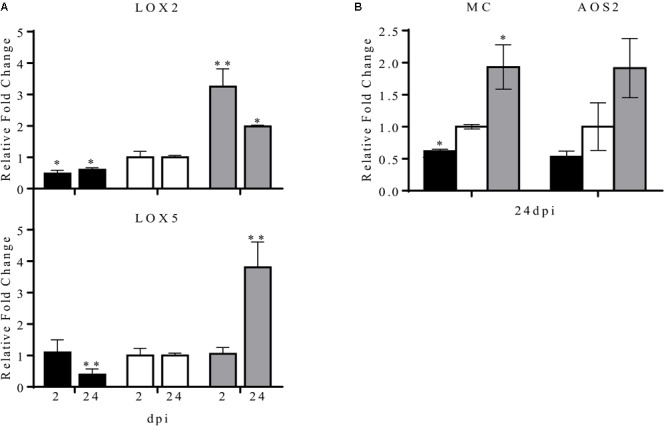
Effect of the modulation of miR319d/TCP10 expression in JA-biosynthetic and JA-responsive genes expression. Transgenic hairy roots from OE319d (black histograms), EV (white histograms) and STTM319 (gray histograms) rhizobia-inoculated plants were collected at 2 and 24 dpi and total RNA was isolated for qRT-PCR analysis. Expression analysis of the JA-biosynthetic genes *LOX2, LOX5* **(A)** and *AOS2* and of the JA-responsive gene *MC* **(B)**. Values were normalized to the values of the EV-transformed control plants that was set to one. Values represent means ± SD from three biological replicates and two technical replicates each. The Mann–Whitney null hypothesis statistical test is relative to EV control data (^∗^ and ^∗∗^ represent a *p-*value < 0.05 and *p-*value < 0.01, respectively).

In *P. vulgaris* at least five *LOX* genes have been identified, these genes differ in their expression pattern in different plant organs. Of these, the *LOX2* and *LOX5* genes are expressed during nodule development (Porta and Rocha-Sosa, 2000). In this work we analyzed the common bean *LOX2* and *LOX5* expression level in transgenic nodulated roots with overexpression or silencing of miR319d (**Figure 6**). As compared to control (EV) plants, the expression level of *LOX2* was lower in OE319d plants in contrast to its high level in STTM plants, both at early (2 dpi) and later (24 dpi) symbiotic stages (**Figure 6A**). These data indicate the correlation of LOX2 expression level with the level of TCP10 in transgenic nodulated roots (**Figures 4, 6**). In addition, the expression levels of the JA-biosynthetic genes *LOX5* and *AOS* as well as the *MULTICYSTATIN* (*MC*) JA-responsive gene (Uppalapati et al., 2005; López-Ráez et al., 2010; Martínez-Medina et al., 2017) showed a similar trend to that observed for *LOX2*, being highly expressed in STTM319d nodulated roots at 24 dpi (**Figures 6A,B**).

## Discussion

Small RNAs differentially expressed during nodule organogenesis have been identified in different legumes such as *Medicago truncatula*, soybean (*Glycine max*), *Lotus japonicus* and common bean (Lelandais-Brière et al., 2009; De Luis et al., 2012; Turner et al., 2012; Formey et al., 2014, 2015, 2016). However, only few in-depth studies that evidence the role of miRNAs in the rhizobial infection, nodulation or SNF processes have been reported (reviewed by Lelandais-Brière et al., 2016; Li et al., 2017). Our group has demonstrated the participation of common bean miR398b and miR172c in different stages of the rhizobia symbiosis (Naya et al., 2014; Nova-Franco et al., 2015). In this work, we identified common bean miR319d as an important regulator of the rhizobial infection and nodulation.

The conserved miR319 family and its targets TCP TF have been extensively characterized in several plant models but most of these studies have been focused in their contribution to the aerial parts, especially leaf development (Schommer et al., 2012, 2014; Koyama et al., 2017). There are no previous studies about the participation of the miR319/TCP node in the control of the legume – rhizobia SNF symbiosis. The miR319d, one of the nine isoforms identified in the common bean, was highly expressed in nodules with respect to other plant organs and was included in a weighted correlation network together with other miRNAs known as regulators of the rhizobial symbiosis (Formey et al., 2015). Here we evidenced that *TCP10* (Phvul.005G067950), previously identified through degradome analysis (Formey et al., 2015), is the target gene of miR319d. Our data on *TCP10* expression profile validated those from the *P. vulgaris* Gene Expression Atlas (O’Rourke et al., 2014) and from the Phytozome data base^5^, regarding the negative correlation with miR319d expression in fertilized roots vs. inoculated roots/nodules at different stages of the symbiosis (**Figure 1**) and in roots/nodules from composite plants overexpressing or silencing the function of miR319d (**Figure 4**).

**FIGURE 7:**
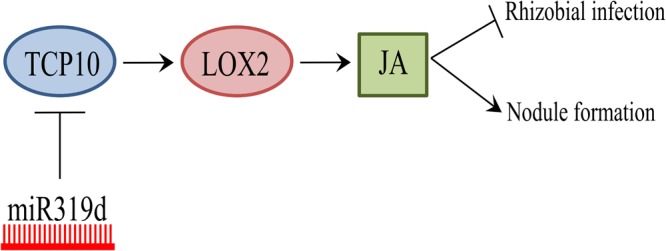
Model of miR319d/TCP10 regulation in the common bean-rhizobia symbiosis. Positive regulation is represented with arrows and negative regulation with lines. A high level of miR319d induces TCP10 degradation (blue circle) that positively regulates the promoter activity of *LOX2* gene (red circle) from the JA biosynthetic pathway. JA (green square) has a negative effect in rhizobial infection but a positive effect of nodule development in common bean.

The regulatory role miR319/TCP in leaf development has been linked with other processes such as the control of cell proliferation by GRF TFs targets of miR396 (Nicolas and Cubas, 2016). However, our data do not support the link of TCP10 as activator of miR396 in inoculated common bean roots (**Supplementary Figure S4**). The regulation of the miR319/TCP node has also been linked to JA signaling that controls different developmental processes such as senescence (Nicolas and Cubas, 2016). JA, along with other phytohormones like ethylene and cytokinin, is a signaling molecule involved in leaf senescence and other developmental processes. The binding of the Arabidopsis TCP TFs TCP4, the target of miR319, and the class I TCP20 to specific motifs within the promoter regions of the *LOX2* JA-biosynthetic gene has been demonstrated through electrophoretic mobility shift and chromatin immunoprecipitation analyses (Schommer et al., 2008; Danisman et al., 2012). In agreement, Arabidopsis *LOX2* is one of the most affected genes depending on TCP4 levels (Schommer et al., 2008, 2012). On this basis, subsequent research in different plant species (i.e., rice, cotton) has linked the TCP transcriptional regulation to JA signaling, through the direct effect in *LOX2* expression, that results in modulation of other JA-related genes (Hao et al., 2012; Zhang et al., 2016). In this work we showed a correlation between *LOX2* and *TCP10* (target of miR319d) transcript levels in transgenic roots with miR319d over-expression or function silencing (**Figure 6**). The latter indicates that, in common bean roots, *LOX2* may be transcriptionally regulated by TCP10, a hypothesis also supported by the identification of TCP TF binding sites (TFBS) statistically over-represented (*p-*value < 0.05) within the *LOX2* 5′-promoter region. In addition, we observed a correlation of the transcriptomic response of other JA-related genes (*LOX5, MC, AOS2*) with TCP10 and LOX2 levels (**Figure 6**). Based on this correlation, we propose that the effect of miR319d/TCP10 node in common bean root growth and nodulation, reported here, may be mediated by JA signaling (**Figure 7**).

The root growth inhibition was one of the first physiological effects detected for JA (Dathe et al., 1981; Staswick et al., 1992; Wasternack, 2007). Previous reports from Arabidopsis relate elevated JA levels with reduced root growth (Ellis et al., 2002; Wasternack, 2007). In this work we showed decreased biomass and area of common bean roots from miR319d-silenced plants with higher expression of *TCP10* and *LOX2* genes (**Figures 4, 6** and **Supplementary Figure S2**).

Several studies have shown the participation of JA as a regulatory/signaling molecule in the rhizobia symbiosis with different legume species, including common bean (Poustini et al., 2007; Ferguson and Mathesius, 2014). For example, there are reports showing negative JA effects in early symbiotic stages of *L. japonicus*- and *M. truncatula* – rhizobia symbioses (Nakagawa and Kawaguchi, 2006; Sun et al., 2006) as well as positive JA effects in soybean nodulation (Seo et al., 2006; Kinkema and Gresshoff, 2008). Here, we showed that the *R. tropici*-inoculated OE319d common bean plants, with low level of TCP10 and LOX2, exhibit a significant increase in the amount of root hair deformation and infection thread formation at early stages of the symbiosis but a decreased nodulation. These results indicate an arrested infection, after infection thread formation stage, that prevents nodule development (**Figures 5–7**). We propose that the regulation of common bean nodulation by miR319d/TCP10 could be mediated by JA signaling. However, because of the complex and intricate regulation of the rhizobia symbiosis we cannot rule out the participation of other signaling pathways in the affected nodulation of common bean plants modulated in the expression of miR319d/TCP10. This is the first report about the miR319/TCP node as regulator of the rhizobial symbiosis, future in-depth studies would indicate the commonalities of such regulatory network in other legume species.

## Author Contributions

JM-R and DF conceived and performed the experiments, interpreted the data and contributed to the drafting of the manuscript. AL performed the experiments. GH conceived and supervised the whole project and wrote the manuscript.

## Conflict of Interest Statement

The authors declare that the research was conducted in the absence of any commercial or financial relationships that could be construed as a potential conflict of interest.

## Footnotes

1 https://phytozome.jgi.doe.gov/pz/portal.html

2 www.mirbase.org

3 www.phytozome.net

4 www.phytozome.net

5 www.phytozome.net
