# Supplementary material for: The MicroRNA319d/TCP10 Node Regulates the Common Bean – Rhizobia Nitrogen-Fixing Symbiosis
Source: Front Plant Sci. 2018 Aug 10;9:1175. doi: 10.3389/fpls.2018.01175 (PMC6095992; doi:10.3389/fpls.2018.01175)
Supplement: Supplementary file 3 [file Table_3.DOCX]

Table S3. Expression analysis of the early symbiosis marker gene ENOD40 and nitrogenase activity of *R. tropici*-inoculated common bean plants.

| **R. tropici inoculated plants** | **Expression level ENOD40** | | | **Nitrogenase Activity**  **(nmol ethylene h-1/Nodules DW)** |
| --- | --- | --- | --- | --- |
|  | Roots-Fertilized | Roots-Inoculated | Nodules |  |
| **3dpi** | 4.46 ± 0.62 | 40.85 ± 17.62 |  |  |
| **10dpi** | 5.20 ± 0.66 | 58.28 ±37.02 |  |  |
| **15dpi** | 1.22 ± 0.14 | 123.53 ± 17.39 | 160.27 ± 18.70 | 66.80 ± 19.50 |
| **21dpi** | 1.10 ± 0.14 | 70.12 ± 28.55 | 112.80 ± 14.69 | 121.60 ± 11.90 |
| **35dpi** | 1.09 ± 0.13 | 7.83 ± 1.14 | 23.85 ± 1.45 | 60.30 ± 12.00 |

The expression levels of common bean *ENOD40* gene were determined by qRT-PCR in roots and nodules from *R. tropici* CIAT 899-inoculated plants at the indicated days post inoculation (dpi). For comparison, ENOD40 expression levels were also determined in roots from fertilized (non-inoculated plants). Expression level refers to gene expression, based on Ct value, normalized with the expression of the housekeeping *UBC9*, *HSP* and *MDH*. Values represent means ± sd from three biological replicates and two technical replicates each. Nitrogenase activity assayed by the acetylene reduction, were determined in roots with nodules harvested at different days post inoculation from *R. tropici* CIAT 899-inoculated plants. Values are expressed in nmol ethylene h-1/Nodules DW, these represent the means ± sd of eight to ten biological replicates.
