# Supplementary figures and images for: The MicroRNA319d/TCP10 Node Regulates the Common Bean – Rhizobia Nitrogen-Fixing Symbiosis
Source: Front Plant Sci. 2018 Aug 10;9:1175. doi: 10.3389/fpls.2018.01175 (PMC6095992; doi:10.3389/fpls.2018.01175)

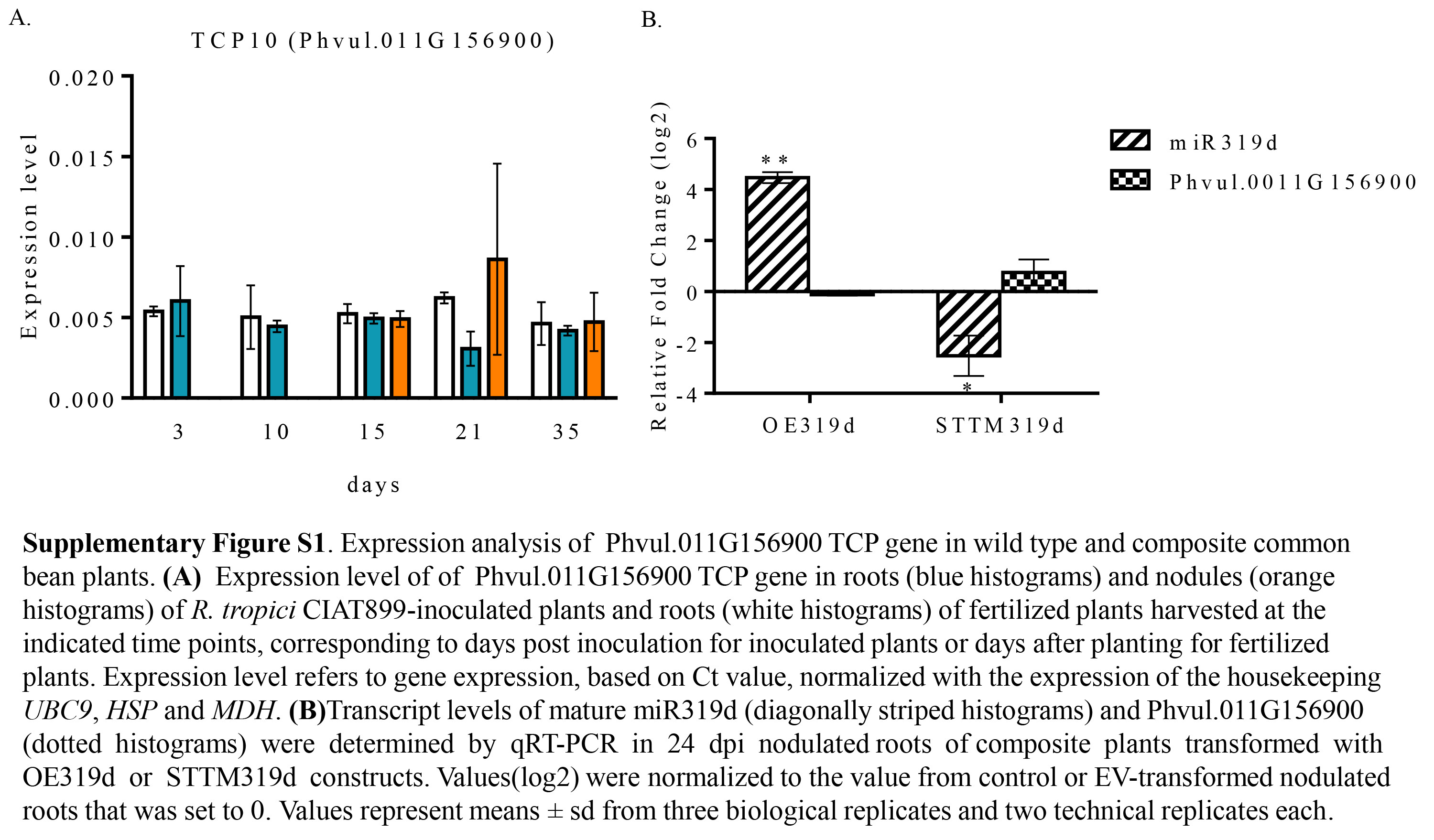

Supplement: Supplementary file 4 [file Image_1.JPEG]

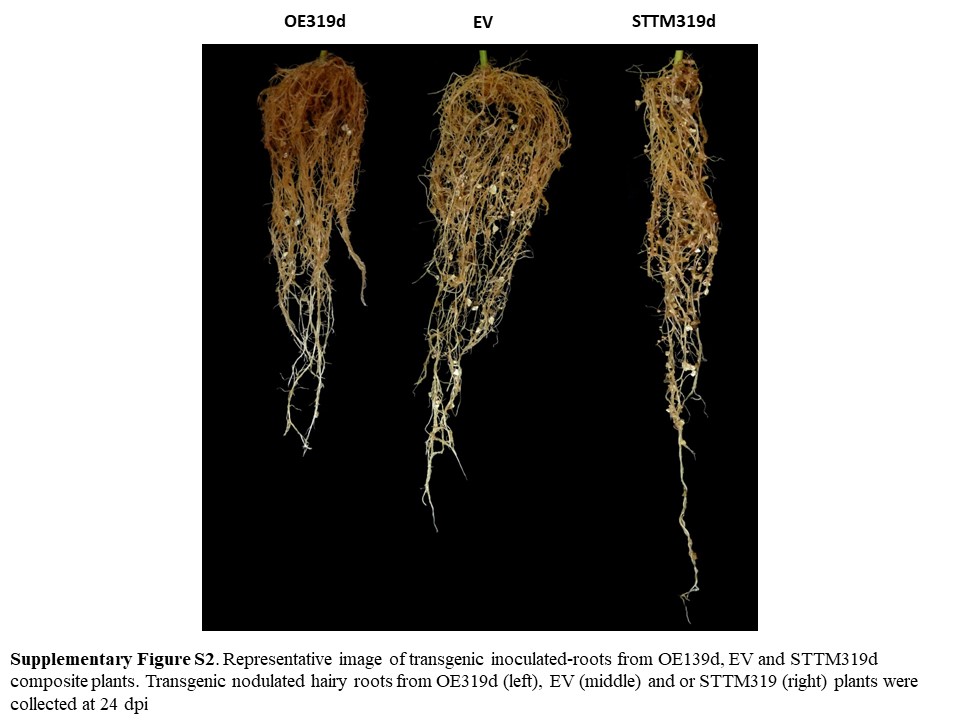

Supplement: Supplementary file 5 [file Image_2.JPEG]

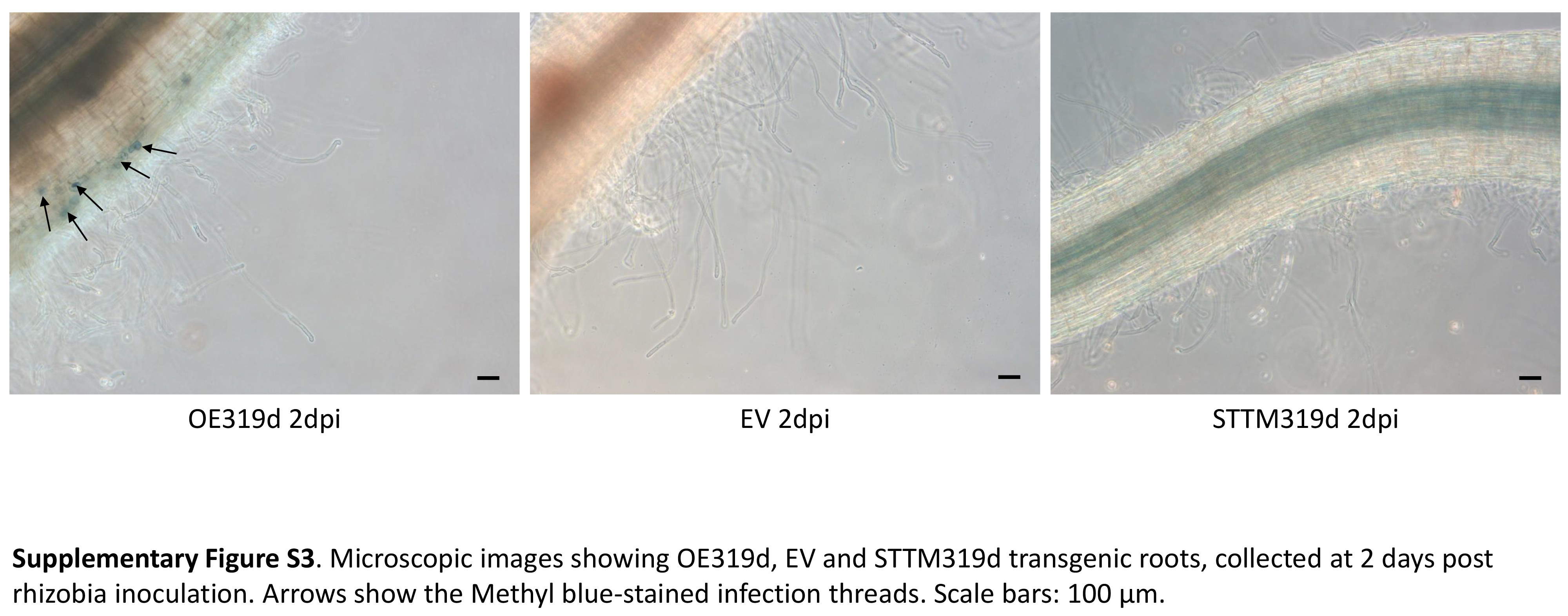

Supplement: Supplementary file 6 [file Image_3.JPEG]

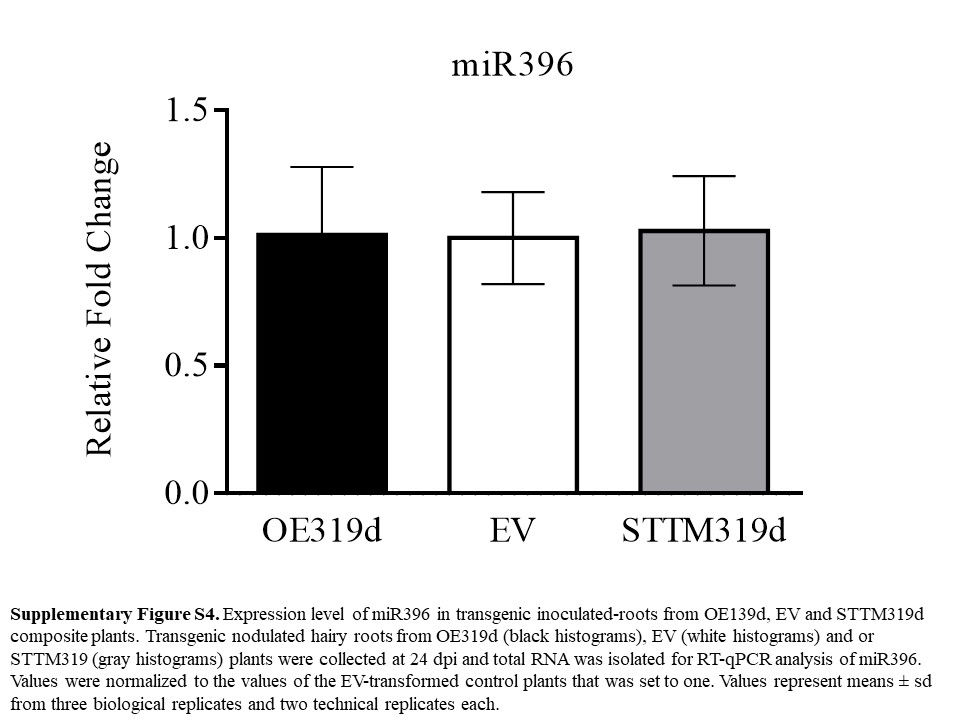

Supplement: Supplementary file 7 [file Image_4.JPEG]
